# Storage of multiple single-photon pulses emitted from a quantum dot in a solid-state quantum memory

**DOI:** 10.1038/ncomms9652

**Published:** 2015-10-15

**Authors:** Jian-Shun Tang, Zong-Quan Zhou, Yi-Tao Wang, Yu-Long Li, Xiao Liu, Yi-Lin Hua, Yang Zou, Shuang Wang, De-Yong He, Geng Chen, Yong-Nan Sun, Ying Yu, Mi-Feng Li, Guo-Wei Zha, Hai-Qiao Ni, Zhi-Chuan Niu, Chuan-Feng Li, Guang-Can Guo

**Affiliations:** 1 Key Laboratory of Quantum Information, University of Science and Technology of China, CAS, Hefei, Anhui 230026, China; 2 Synergetic Innovation Center of Quantum Information and Quantum Physics, University of Science and Technology of China, Hefei, Anhui 230026, China; 3 The State Key Laboratory for Superlattices and Microstructures, Institute of Semiconductors, CAS, PO Box 912, Beijing 100083, China

## Abstract

Quantum repeaters are critical components for distributing entanglement over long distances in presence of unavoidable optical losses during transmission. Stimulated by the Duan–Lukin–Cirac–Zoller protocol, many improved quantum repeater protocols based on quantum memories have been proposed, which commonly focus on the entanglement-distribution rate. Among these protocols, the elimination of multiple photons (or multiple photon-pairs) and the use of multimode quantum memory are demonstrated to have the ability to greatly improve the entanglement-distribution rate. Here, we demonstrate the storage of deterministic single photons emitted from a quantum dot in a polarization-maintaining solid-state quantum memory; in addition, multi-temporal-mode memory with 1, 20 and 100 narrow single-photon pulses is also demonstrated. Multi-photons are eliminated, and only one photon at most is contained in each pulse. Moreover, the solid-state properties of both sub-systems make this configuration more stable and easier to be scalable. Our work will be helpful in the construction of efficient quantum repeaters based on all-solid-state devices.

## Abstract (web summary)

Quantum repeaters are critical components for distributing entanglement over long distances, and they can be improved by the elimination of multi-photon-pair events. Here, the authors demonstrate the storage of single photons emitted by a quantum dot in a polarization maintaining solid-state memory.

## Graphical abstract

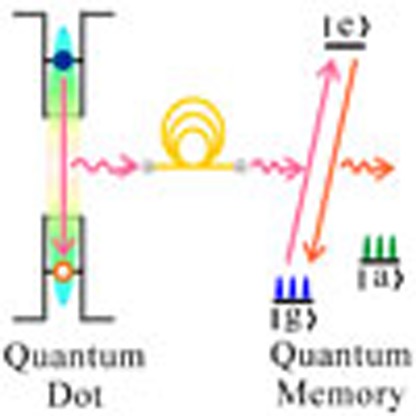

Long-distance entanglement distribution has become increasingly important, which is essential in the improvement of many quantum technologies, such as quantum key distribution^1^ and quantum internet^2^. It is also helpful in the examination of the foundation problems in quantum mechanics, for example, the Bell inequality test^3^. However, this task is not easy to perform, because of the photon loss during the fibre transmission. One proposal to overcome this issue is to use quantum repeaters^4^. In this architecture, the entire distance is divided into several shorter elementary links, and for each link, entanglement between quantum memories can be established independently. Next, the elementary links are joined using entanglement swapping to create an entangled pair over the entire distance.

The first concrete quantum repeater proposal is the well-known Duan–Lukin–Cirac–Zoller (DLCZ) protocol^5^, in which the atomic ensembles are used as the quantum memories and the photon sources. However, the entanglement-distribution rate is still quite low in this scheme. To solve this problem, many improved architectures of quantum repeaters have been proposed^6^,^7^,^8^,^9^,^10^. Of these proposals, the main approaches include the single-photon source^7^ (or the single entangled photon-pair source) and the multimode quantum memory^6^,^9^ and so on. Using a single-photon source, the multiphoton errors can be eliminated, which makes the photon emission rate greatly enhanced, thus improving the entanglement-distribution rate. Meanwhile, the multimode memory protocol has been estimated to be the most efficient protocol among all the quantum repeater architectures^11^.

On one hand, quantum dots (QDs) are attracting increasing interest in the quantum memory research community, and notable recent work includes the slowing-down experiment of QD emissions in the hot atomic ensemble^12^, and the absorption of QD emissions in a single ion^13^. This increasing interest is primarily because of the potential of QDs to be a good source of deterministic single photons^14^,^15^ and also a good source of deterministic entangled photon pairs^16^. On the other hand, multimode quantum-memory protocols are being intensively studied. Many degrees of freedom of the photons, such as time bins^17^,^18^,^19^,^20^,^21^, frequencies^9^, polarizations^21^,^22^,^23^,^24^,^25^, spatial directions^26^ and orbital angular momentum modes^27^, can be used to multiplex the memory and the communication channel. Time bins are one type of the most-used modes. Rare-earth (RE)-ion-doped solids have the advantage of broad inhomogeneous absorption, which provides a large memory bandwidth for these solid-state quantum memories^19^, and enables them to be used for temporal multimode operations.

In this work, we experimentally demonstrate two points. The first point is the storage of deterministic single photons (with no multi-photons in principle) emitted from a semiconductor self-assembled QD in a solid-state polarization-maintaining quantum memory^24^, which is based on Nd^3+^:YVO_4_ crystals. The QD and the RE-ion-doped crystals are separated by 5 m on two separate optical tables and are connected via a 10-m fibre (see Fig. 1). The second point is the realization of the temporal multiplexed quantum memory with QD-based narrow single-photon pulses. 1, 20 and 100 temporal modes are respectively stored in the quantum memory, with at most one photon present in each mode. Both of these points will be helpful in the development of quantum repeaters. Moreover, both sub-systems in our experiment are solid state, which will make this configuration more stable and convenient.

## Results

### Wavelength matching between QD and the quantum memory

The absorption line of the Nd^3+^:YVO_4_ crystal is located at 879.7 nm, with a narrow memory bandwidth of hundreds of MHz, whereas the emission lines of InAs/GaAs QDs are random, depending on the dot size and the chemical composition in a wide range of approximately 900∼1,200 nm. 879.7 nm is an embarrassing wavelength because it is far away from the emission range of QDs. Moreover, the wetting-layer emission of the InAs/GaAs QD sample is located at ∼860 nm, with a full width at half maximum of tens of nanometres, and is much more intense than the signal photons. This emission represents a strong noise source at 879.7 nm. To solve this problem, we employ three steps, including QD sample fabrication with a special design (blue-shift the QD emissions to around the memory band, see Methods and Supplementary Note 1 for details, and the sample structure is shown in Supplementary Fig. 1), QD selection (select an emission nearest to the memory band, see Methods) and local heating of the selected QD with a strong laser (finely tune the QD emission to the memory band, see Methods), to derive a QD emission that matches the memory band of the quantum memory.

Figure 2 shows the experimental results. Figure 2a is the photoluminescence spectrum of a QD, which is sifted out from hundreds of QDs on this specially designed QD sample, namely, the result of the first and second steps. The reason for selecting this QD is that its emission line is at the wavelength of 879.5 nm, which is slightly shorter than the wavelength of the memory band. However, no other lines are detected in the region. This result may be caused by the unbalanced carrier capture (this process may cause the generation of trion, which can be examined by the vanish of fine structure splitting^28^,^29^) and the filtering effect of the planar distributed Bragg reflector (DBR) microcavity (see Supplementary Note 2; Supplementary Fig. 2 for details). Figure 2b shows the local-heating spectrum for this QD (see Supplementary Note 3 for local heating, and the sketch of the local heating and some additional data are shown in Supplementary Fig. 3). When the power of the 910-nm heating laser reaches 24 mW, the emission line can be finely red-shifted to 879.7 nm (ref. 30), that is, the memory band. The autocorrelation of this emission line is measured to be ∼*g*^(2)^(0)=0.14, which demonstrates that the photons were emitted from a good-quality single-photon source.

### Storage for single photons

In our experiment, the quantum memory is based on the atomic frequency comb (AFC) protocol, according to which the absorption band of the Nd^3+^-ion ensemble in the crystals (see Methods for the details of the quantum-memory sample) must be tailored into a frequency comb. The period of the frequency comb (denoted as Δ) determines the storage time of the single photons (*T*_storage_), and the range of the frequency comb determines the memory bandwidth. The details about the AFC quantum memory technique are presented in the Methods and in Supplementary Note 4, and a sketch is shown in Supplementary Fig. 4.

**Figure 1:**
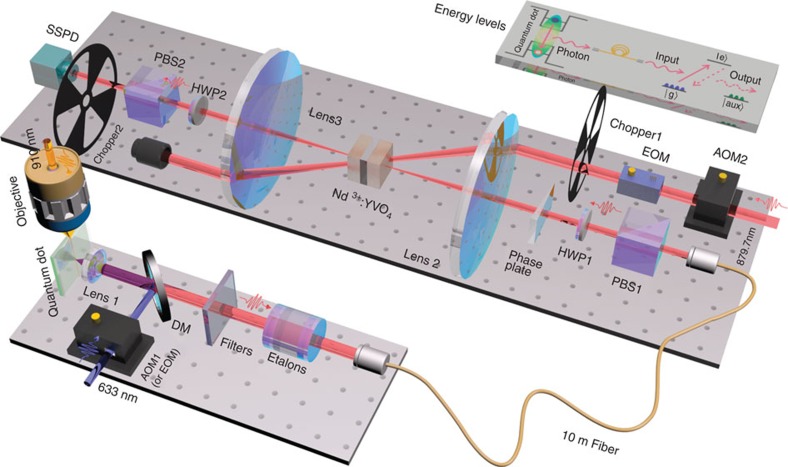
Experimental set-up. This experiment is performed on two separated optical tables connected by a 10-m-long optical fibre. The QD sample and the Nd^3+^:YVO_4_ crystals are spaced 5 m apart. A single QD embedded in a planar DBR microcavity is excited by a 633-nm laser, and a 910-nm laser is used to shift the wavelength of the QD emission using the local-heating effect. The QD emission is precisely shifted to the ^4^I_9/2_→^4^F_3/2_ transition of Nd^3+^ with the calibrated etalon. The 879.7-nm laser, which is modulated by AOM2 and electro-optic modulator (EOM) both in intensity and in frequency, is used to pump the Nd^3+^:YVO_4_ crystals to create a frequency comb according to the AFC protocol. The single photons are then stored in the crystals and retrieved after a time *T*_storage_. An SSPD with a low dark count is used to detect the single photons. PBS1, HWP1, PBS2, HWP2 and the phase plate are used to prepare and measure the polarization qubit when the qubit-memory experiment is performed. The arrows indicate the directions of the light beams. The AOMs (or EOM used for modulating excitation light) and the choppers are synchronized to an electrical-pulse generator to create the time sequence for this experiment. The inset shows the energy levels of the QD and the Nd^3+^-ions. The QD, which can contain an exciton, biexciton or a trion, emits a single photon, which is then sent to the quantum memory via a fibre, and is subsequently absorbed by the ion ensemble with a frequency comb. After the storage time, the photon is re-emitted by the ions. |*g*〉, |*e*〉 and |*aux*〉 denote the ground, excited and auxiliary levels of Nd^3+^ ions, respectively.

**Figure 2:**
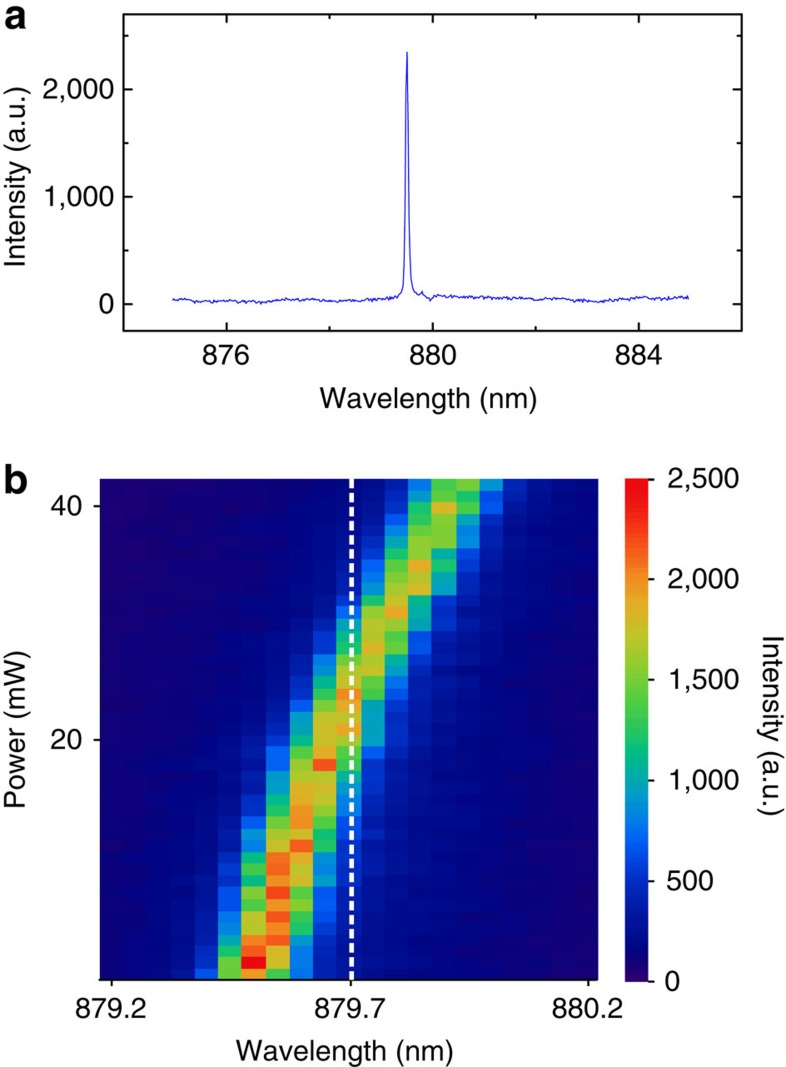
Photoluminescence spectra. (**a**) Photoluminescence spectrum without local heating. A single peak appears at the position of 879.5 nm, which may be caused by the unbalanced carrier capture and the filtering effect of the DBR microcavity (see Supplementary Note 2 for the explanations). The wavelength of this peak is a little shorter than that of the memory band (879.7 nm), which provides the chance to tune it to match the memory band using a local-heating effect. (**b**) Power-dependent spectra. The *x* axis is the wavelength of QD emission, and the *y* axis is the power of the 910-nm laser, which is used for local heating. The colour represents the photoluminescence intensity of the QD emission. The peak is shifted across 879.7 nm with increasing laser power. When the power reaches 24 mW, the emission line matches the memory band.

**Figure 3:**
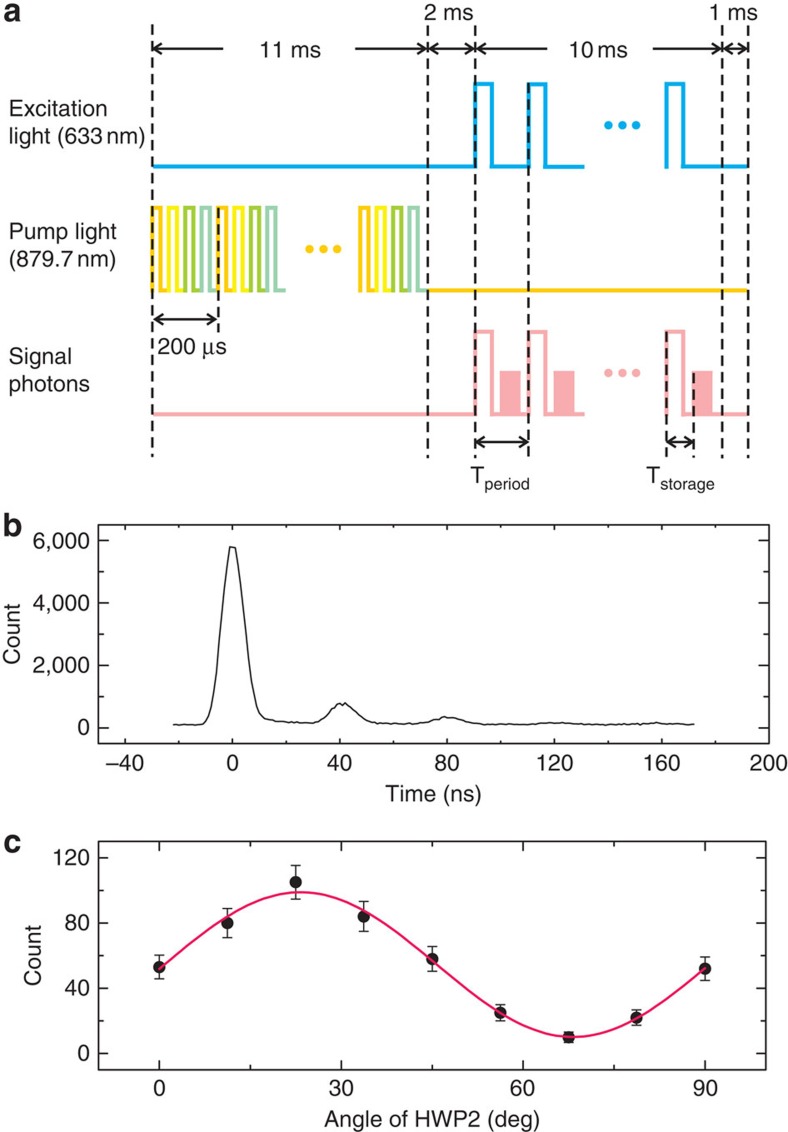
Time sequence and experimental results. (**a**) Time sequence diagram. The entire procedure includes a 11.5-ms preparation time, a 2.5-ms wait time, a 10-ms storage and retrieval time and another 1-ms wait time. During the preparation time, the pump pulses with different frequencies (represented by different colours) are used to pump the Nd^3+^:YVO_4_ crystals to prepare the AFC (see Supplementary Note 4 for methodological details). In the storage and retrieval procedure, the pump laser is blocked, and then the excitation light associated with the single photons is modulated to a series of pulses with a period of *T*_period_. After a storage time *T*_storage_, the signal photons are retrieved. (**b**) Time spectrum of the stored single photons. The storage time is 40 ns, and the pulsewidth of the excitation light is 10 ns. The first peak represents the light that is not absorbed. The second peak represents the retrieved single photons, and the third peak is the second-order retrieved photons. (**c**) Polarization-qubit quantum memory. Single photons are encoded with a qubit |*H*〉+|*V*〉 and then sent to the quantum memory. The storage time is 40, and a 2-ns coincidence window is chosen for the retrieved single photons. The retrieved qubit is projected to a series of bases, which are represented by the angles of HWP2. The count shows a sinusoidal oscillation with a fidelity of 0.913±0.026. The error bars in these data are due to the counting statistics, namely, the standard deviation.

Figure 3b shows the experimental result of the storage of the single photons. To obtain this time spectrum, the storage and retrieval period (*T*_period_) and the pulsewidth of the excitation pulse (*T*_expw_, which is related to the signal pulsewidth) are set to be 400 and 10 ns, respectively. The storage time is *T*_storage_=40 ns, and the integration time is 2.8 h. The peak at 0 ns corresponds to the single photons that are not absorbed as well as some background wetting-layer light, the peak at 40 ns corresponds to the single photons that are stored and retrieved and the peak at 80 ns is the second-order retrieved single photons. The second peak is relatively small compared with the first peak. This result is related to another merit of the quantum memory, which can be used as a good narrow-bandwidth filter^31^. The single photons are filtered by the filter and etalon (Fig. 1) before they are sent to the quantum memory. However, the background wetting-layer light can pass through the etalon from its other transmission peaks (although every copy of the leaked wetting-layer light is little, there are many copies of them). Next, we use the quantum memory itself as a filter to make the single photons clear. Therefore, the retrieved signal contains only single photons, and the memory efficiency is not as low as that shown in this figure; this point will be discussed later. The signal-to-noise ratio (SNR) of the stored photons is estimated to be 9:1. Approximately 25% of the noise stems from the dark counts of the superconducting single-photon detector (SSPD), 25% comes from the pump laser and 50% stems from the ambient light.

Our sandwich-like quantum memory has been demonstrated to be polarization-maintaining. Process tomography has shown that its fidelity can reach 0.999±0.002 using the attenuated laser^24^. Here, we take a typical case as an example to show the preservation of quantum coherence during the storage and retrieval process, which is important in quantum applications (including quantum repeater). Figure 3c shows the experimental result. The error bars in these data are due to the counting statistics. The deterministic single photons are encoded with a quantum state of |*H*+*V*〉 by PBS1 (polarizing beamsplitter) and HWP1 (half-wave plate, see Fig. 1; the phase plate is used to compensate for the phase difference induced by the crystals) and are then sent to the quantum memory (see Methods for sample structure and ‘*H*', ‘*V*' definitions). After a storage time of *T*_storage_=40 ns, the single photons are retrieved. The polarization characteristics of these retrieved photons are examined via HWP2 and PBS2. A 2-ns window is selected at the position of 40 ns, and the integration time is 1,000 s. As the angle of HWP2 is rotated from 0° to 90°, we observe a sinusoidal oscillation of the single-photon counts. The maximum value appears at the position of 22.5° (|*H*+*V*〉 basis), and the minimum value appears at 67.5° (|*H*−*V*〉 basis) of HWP2. The fidelity of the retrieved polarization state is estimated to be 0.913±0.026 (this value is also directly associated with the fidelity of the storage process). These results show that the |*H*+*V*〉 state can be well preserved.

### Storage of multiple single-photon pulses

To further reduce the photon numbers in a single pulse while increasing the mode numbers of the photons that can be stored, we use an electro-optic modulator to replace AOM1 (acousto-optic modulator; see Fig. 1). The pulsewidth of the excitation laser *T*_expw_=0.8 ns is reduced to be less than the QD's lifetime, which ensures there is only one photon in a single pulse. This point is also demonstrated by the Hanbury Brown–Twiss experiment (see details in Supplementary Note 5 and Supplementary Fig. 5).

**Figure 4:**
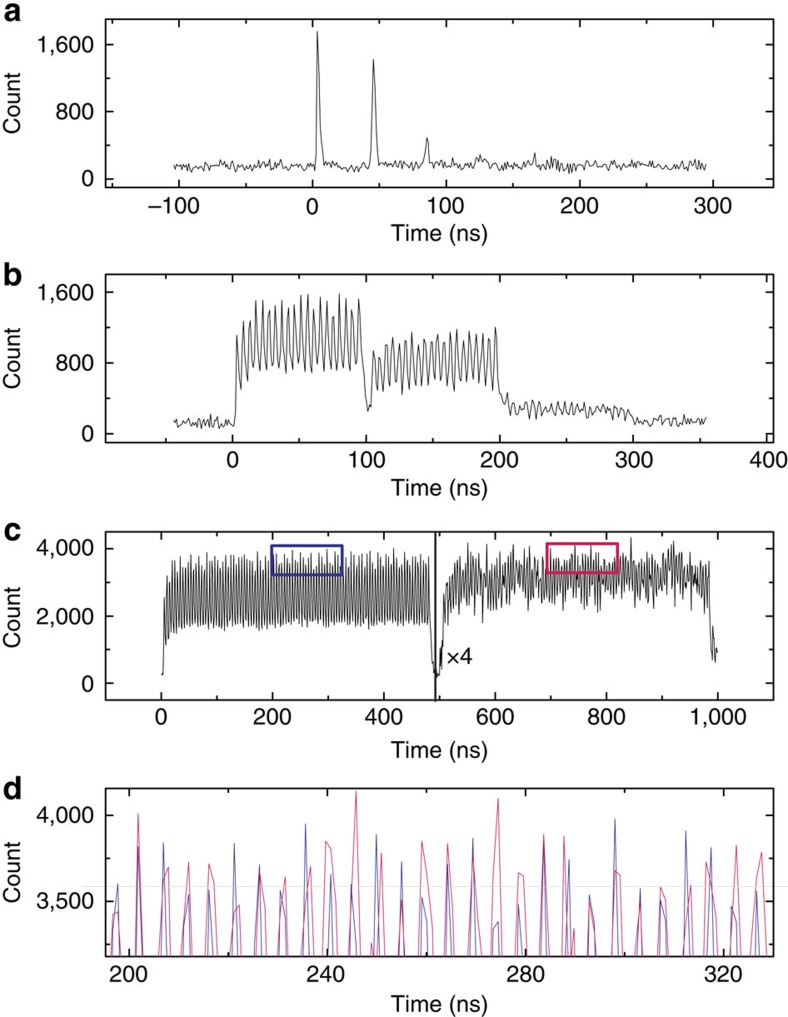
The quantum storage of multiple single-photon pulses. We use an electro-optic modulator to modulate the excitation light here, and its pulsewidth *T*_expw_ is reduced to 0.8 ns, which ensures there is at most one photon in each pulse. (**a**) 1, (**b**) 20 and (**c**) 100 temporal modes of the single photons are used for the quantum memory. The first group of peaks represents the transmitted photons, second group of peaks represents the stored single photons and the third group of peaks (not in **c**) represents the second-order retrieved photons. The storage times are 40, 100 and 500 ns, respectively. In **c**, the counts of the second group of peaks are multiplied by a factor 4. (**d**) The enlargement of the rectangle regions in **c**. The blue peaks correspond to the transmitted-photon signals in the blue rectangle, and the pink peaks correspond to the stored-photon signals in the pink rectangle, but with the time coordinate subtracted by 500 ns (the storage time). By comparing these two groups of peaks, we find that each of the peaks in the transmitted and stored signals correspond to each other well. This result shows that the temporal modes of the single photons are well maintained during the memory process.

Figure 4a shows the result of the storage for 1 single-photon pulse with *T*_period_=400 ns and *T*_storage_=40 ns. The integration time is 11.7 h. The peak at 0 ns corresponds to the single photons that are not absorbed without wetting-layer light, the peak at 40 ns corresponds to the single photons that are stored and the peak at 80 ns is the second-order retrieved single photons. An additional set of filter and etalon (with a different free spectral range) are inserted in the beampath here to filter the single photons more clearly. In this situation, the second peak is almost as high as the first peak. In Fig. 4b, 20 single-photon pulses are stored in the quantum memory with *T*_period_=400 ns and *T*_storage_=100 ns. The integration time is 7.5 h, and the separation between the neighbouring modes is 4.8 ns. Twenty peaks are clearly seen in the range of 100∼200 ns, which are the stored single-photon temporal modes. The peaks in the ranges of 0∼100 and 200∼300 ns are the transmitted light and the second-order retrieved light, respectively.

We also examine the situation of 100 modes with *T*_period_=1,000 ns and *T*_storage_=500 ns, as shown in Fig. 4c. The integration time and the separation between the neighbouring modes are 46.1 h and 4.8 ns, respectively. The peaks in the ranges of 0∼500 and 500∼1,000 ns are the transmitted light and the retrieved single photons, respectively. In fact, the efficiency of quantum memory is related to the storage time. In the present situation, the efficiency is ∼7%, whereas this value is estimated to be 20 and 13% in the situations of *T*_storage_=40 ns and *T*_storage_=100 ns, respectively. In spite of the decrease in the memory efficiency, we can still clearly observe 100 small peaks from the retrieved photons. Figure 4d shows the details of the peaks in the blue rectangle in Fig. 4c and those in the pink rectangle with the time coordinate subtracted by *T*_storage_=500 ns. Each of these peaks corresponds well to each other one by one. This phenomenon shows the reliability of our experimental results.

## Discussion

In our work we demonstrate the storage of QD-based deterministic single-photon pulse trains. Both the sub-quantum systems in this configuration, namely, the QD and the RE-ion-doped crystals, are solid-state materials. Moreover, we demonstrate that the polarization states of the single photons can be well preserved. Therefore, both the polarization states and the time bins can be used to encode the qubits. Notably, although the single-photon case is described here, the QD also has the potential to be a high-quality deterministic entangled photon-pair source or a solid spin qubit that is entangled with a single photon^32^. In these situations, our configuration could be conveniently utilized. All of these characteristics make our configuration more suitable for the construction of the efficient quantum repeaters.

One possible application of our configuration is the quantum repeater protocol recently proposed by Sinclair *et al.*^9^, which is based on spectral multiplexing, multimode AFC delay quantum memory, entangled photon-pair sources, Bell-state measurement and feed-forward control. This protocol can, on one hand, decrease the requirement of the on-demand quantum memory, and on the other hand, greatly improve the entanglement-distribution rate because the connections of the elementary links are processed simultaneously. The hierarchical connections are avoided in this protocol, and thus, long-distance classical communications are also avoided. In our experimental configuration, temporally multiplexed quantum memory has been demonstrated (current work), and spatially multiplexed memory (orbital angular momentum modes) has also been demonstrated to be realizable^27^. Moreover, the QD has been revealed to possess the ability to be a narrow-linewidth indistinguishable deterministic entangled photon-pair source^16^. Therefore, our work makes us one step closer to this high-efficiency quantum repeater architecture. Moreover, the determinacy property of the QD emissions also plays a crucial role for the improvement of the entanglement-distribution rate in this protocol, which has been calculated in detail in further work by Guha *et al.*^33^

Another example of the application of our configuration is the quantum repeater protocol based on single-photon sources^7^, which improves upon the DLCZ protocol by replacing the photon-pair sources (an equivalent protocol as the DLCZ one) with single-photon sources. In the former case, to avoid the errors caused by the multi-photon-pair emissions, the emission rate of the photon pairs must be greatly limited, whereas in the latter case, this problem is eliminated. Therefore, the entanglement-distribution rate can be improved. In our work, the single-photon storage can be utilized in this protocol (when spin-wave AFC memory is integrated), and furthermore, by combining the multi-temporal-mode quantum memory^6^, the quantum repeater can be faster and more robust.

Our work can probably be used in other quantum technologies, for example, the quantum networks^2^,^34^,^35^and so on. Although the quantum information flowing between QD and the quantum memory is not demonstrated here, the quantum channel between them has been demonstrated, and the polarization information can be preserved. The generation of photon-spin entanglement has been demonstrated to be realizable in the QD^32^. Therefore, the exchange of quantum information between the QD and the quantum memory can be in principle achieved.

To conclude, we achieve the storage of deterministic single photons emitted from a QD in a sandwich-like Nd^3+^:YVO_4_ quantum memory, which can preserve the polarization states of the input photons. We have also demonstrated the temporal multimode operation of the quantum memory with 1, 20 and 100 narrow single-photon pulses. Only one photon exists in a single pulse at most. Our work paves the way towards the construction of high-speed quantum repeaters based on all-solid-state devices and can also be used in other quantum technologies.

## Methods

### QD fluorescence collection

A 633-nm laser is used to excite the QD, as shown in Fig. 1. After being reflected by a dichroic mirror, the laser is focused onto the sample by an aspheric lens (Lens 1). The same lens, with a high numerical aperture (NA) of 0.68 and a working distance of 1.76 mm (placed inside the cryostat), is used to collect the fluorescence emitted from the QD. Moreover, the QDs are grown in a planar microcavity comprising a 10-pair Al_0.9_Ga_0.1_As/GaAs DBR on top and a 32-pair DBR on bottom. This microcavity can greatly enhance the collection efficiency of the fluorescence^15^,^36^. The fluorescence is then filtered by the dichroic mirror and a 20-nm bandwidth filter (99% transmission at 879.7 nm and 10^−7^ at 910 nm). When the QD spectra are detected, the fluorescence is sent to a spectrometer (not shown) equipped with a 900-grooves·per mm grating and a Peltier-cooled InGaAs detector array; when the autocorrelation function is measured, the fluorescence is sent to a picosecond time analyzer (not shown). Otherwise, the fluorescence is sent to the subsequent optical elements in Fig. 1.

### Steps for wavelength matching

During the QD sample growth, an AlGaAs layer is grown under the InAs QD because AlAs has a broader energy gap than GaAs, which can blue-shift the QD emissions, as well as the wetting-layer emission. However, problematically, the InAs QD tends to disappear and the quantum efficiency is also substantially reduced when it meets the aluminium-containing layer. Here, we design a special structure of the QD sample, which contains a 20-nm-thick Al_0.2_Ga_0.8_As layer and a 10-nm-thick GaAs layer overlying it (see Supplementary Note 1; Supplementary Fig. 1). The InAs QDs are then grown on the GaAs layer using the molecular beam epitaxy technique. With this special design, the emission lines of QDs are blue-shifted to ∼879.7 nm, and the emissions have a relatively high quantum efficiency and a high SNR. The QD emissions roughly coincide with the ^4^I_9/2_→^4^F_3/2_ transition of Nd^3+^ in the YVO_4_ crystal^24^.

QDs have nonuniform emission wavelengths and emission strength. To find a QD with emission at a wavelength that is slightly shorter than 879.7 nm (the wavelength can only be red-shifted for several 0.1 nm in the following fine-tuning process) and with a high brightness, we place the QD sample on a three-axis nano-positioner with the travel ranges of several millimetres, and the entire set-up is then placed in a low-vibration liquid-helium-free cryostat, where the sample is cooled to 8 K (Montana Instruments). Next, we scan the nano-positioner in the *xy*-plane to detect the QD spectra. After scanning, we compare hundreds of QDs and then identify a single emission line located at the wavelength of 879.5 nm.

To tune this emission line to the Nd^3+^ ion transition exactly, we use a 910-nm laser to perform local heating in the vicinity of the selected QD (see Supplementary Note 3 and Supplementary Fig. 3a). This laser is focused to the cleaved edge of the DBR microcavity using a 50 × long-working-distance objective (see Fig. 1). The microcavity acts as a waveguide for the laser and directs it to the vicinity of the selected QD^36^. The orthogonal geometry and the filter completely eliminate the residual local-heating laser. Compared with global heating, local heating can induce less noise and less inconvenience due to heat expansion. The heating effect can red-shift the emission line of the QD continuously by several 0.1 nm (ref. 30). During this process, a solid etalon (see Fig. 1), which is calibrated by the pump laser of the quantum memory (at 879.7 nm), is used to set the target wavelength of the QD emission. The free spectral range and the bandwidth of the etalon are 50 GHz and 700 MHz, respectively. The position of etalon's transmission line can be adjusted by tuning the temperature of the etalon. When the temperature is set to 38.1 °C, etalon's transmission line is well calibrated to 879.7 nm. The transmission efficiency is greater than 95%. The wavelength of the single photons can be finely tuned to etalon's transmission line by changing the power of the heating laser to 24 mW.

### Quantum memory sample

The sample that we use for the quantum memory is composed of two pieces of nearly identical Nd^3+^:YVO_4_ crystals with a 45° half-wave plate sandwiched between them (see Fig. 1). Each crystal is 3 mm long along the *a* axis and has a doping level of 5 p.p.m.^37^. This sample is cooled to 1.5 K in a liquid-helium-free cryostat (Oxford Instruments, SpectromagPT) with a superconducting magnetic field of 0.3 T parallel to the crystals' *c* axis (the axes of these two crystals are parallel). The ^4^I_9/2_→^4^F_3/2_ transition (∼879.7 nm) of the crystals is used for the quantum memory here, as mentioned earlier. In this transition, the absorption of the |*H*〉-polarized photons in the crystals is much stronger than that of the |*V*〉-polarized photons, with ‘*H*' (‘*V*') denoting the horizontal (vertical) direction, that is, the direction parallel (perpendicular) to the crystals' *c* axis. This sandwich-like structure has been demonstrated to have the ability of maintaining the polarization characteristic of the stored photons, thus enabling the high-fidelity memory of the polarization-based qubit^24^.

### Time sequence and quantum memory set-up

The time sequence is shown in Fig. 3a. During the 11.5-ms preparation time, the frequency of the pump laser (|*H*+*V*〉-polarized so that both crystals can be pumped, see the 879.7-nm laser in Fig. 1) is modulated by AOM2 and an electro-optic modulator to generate a comb-like profile with a bandwidth of ∼500 MHz and a programmable comb period Δ (see Supplementary Note 4 for details). Next, this pump laser is passed through chopper 1, and then focused on the Nd^3+^:YVO_4_ crystals by lens 2 (*f*=250 mm) to prepare the 500-MHz-width AFC. Subsequently, the laser is collimated by lens 3 (*f*=250 mm) and blocked. Meanwhile, an anti-phase chopper (chopper 2) is used to block the residual pump laser to protect the SSPD (Fig. 1). After a 2.5-ms wait time, the 10-ms storage and retrieval procedure begins. The pump laser is blocked by chopper 1, and single photons come out. The modulation of single photons is achieved by modulating the 633-nm excitation laser using AOM1 (Fig. 1). This method can reduce the loss of single photons. The storage and retrieval period is denoted as *T*_period_, and the width of the modulated excitation laser pulse is denoted as *T*_expw_. The single photons hence become a series of pulses as well, with a period of *T*_period_ and a pulsewidth slightly larger than *T*_expw_ due to the QD's lifetime. The single photons are then focused on the Nd^3+^:YVO_4_ crystals by the same lens 2 and in the same site as the pump laser to be absorbed by the frequency-tailored ions. The spot size of the pump laser is tuned to 4 times of that of the single photons, creating good spatial overlap of these two light beams in the non-collinear configuration. This experimental setting sufficiently reduces the noise while maintaining the memory efficiency. Next, after a time of *T*_storage_, which is determined by the period of the frequency comb, the retrieved single photons are re-emitted as a result of the collective interference among all of the ions that are in phase. Finally, the single photons are collimated by lens 3 and detected by the SSPD, which is immersed in liquid helium and operated at 1.5 K. Its detection efficiency for our single photons is ∼8%, and the dark count rate is roughly 1.5 s^−1^. The time spectra are obtained by sending the SSPD signals to a time-correlated single-photon counting system. The electrical pulses that are synchronized with the excitation light pulses (633 nm) are used as the trigger signal. The integration range was chosen to be the same as *T*_period_, and the time interval was chosen to be 1 ns for Fig. 3b and 0.5 ns for Fig. 4.

PBS1, HWP1, PBS2, HWP2 and the phase plate are used for the polarization-qubit-memory experiment, the configurations of which have been introduced in the Results section. HWP2 is rotated to the angle where the photon count is maximized when Figs 3b and 4 are measured. HWP1 does not affect the maximum value of the photon count, but affects that at which angle of HWP2 the maximum count will appear.

### Brief summary about the SNR improvement

First, the QD is grown in a 4*λ* planar DBR microcavity. The vertical mode of this microcavity is centred on ∼879.7 nm (see Supplementary Notes 1 and 2 for details). This structure makes the QD preferentially emit vertically, where the single photons are collected, in contrast with the 4*π* solid-angle emission corresponding to a free QD^36^. Second, we use a high-NA (0.68) aspheric lens (lens 1) placed in the cryostat to collect the single photons instead of a long working-distance objective, which has a typical NA of ∼0.4. The photon count is ∼5 × 10^5^ per second with continue-wave excitation. Third, the intensity modulation is performed on the excitation laser instead of on the single photons directly. This method prevents signal loss during the generation of the time sequence. Fourth, in the quantum-memory experiment, we use a non-collinear configuration for the pump light and the signal photons. This configuration prevents the single-photon loss that occurs during the combination of the signal light beam and the pump light beam, which is required in the collinear configuration. Moreover, the spot size of the focused pump light is four times that of the single photons, allowing the memory efficiency in the non-collinear configuration to be as high as that in the collinear configuration. The non-collinear configuration also greatly suppresses the noise induced by the pump laser. Finally, an SSPD is used instead of a silicon single-photon avalanche diode. The dark count rate of the SSPD can be as low as 1.5 s^−1^.

## Additional information

**How to cite this article:** Tang, J.-S. *et al.* Storage of multiple single-photon pulses emitted from a quantum dot in a solid-state quantum memory. *Nat. Commun.* 6:8652 doi: 10.1038/ncomms9652 (2015).

## Supplementary Material

Supplementary Information Supplementary Figures 1-5, Supplementary Notes 1-5 and Supplementary References
