# Supplementary material for: Storage of multiple single-photon pulses emitted from a quantum dot in a solid-state quantum memory
Source: Nat Commun. 2015 Oct 15;6:8652. doi: 10.1038/ncomms9652 (PMC4667696; doi:10.1038/ncomms9652)
Supplement: Supplementary Information — Supplementary Figures 1-5, Supplementary Notes 1-5 and Supplementary References [file ncomms9652-s1.pdf]

SUPPLEMENTARY FIGURES

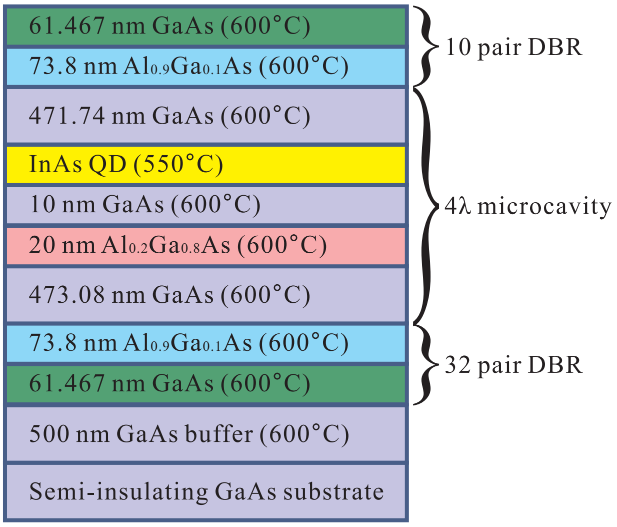

Supplementary Figure 1: **The structure of QD sample.** The 20-nm-thick $Al_{0.2}Ga_{0.8}As$ layer blue shifts the emission of the QD to approximately 879.7 nm. The InAs QD is grown in a 4λ planar DBR microcavity.

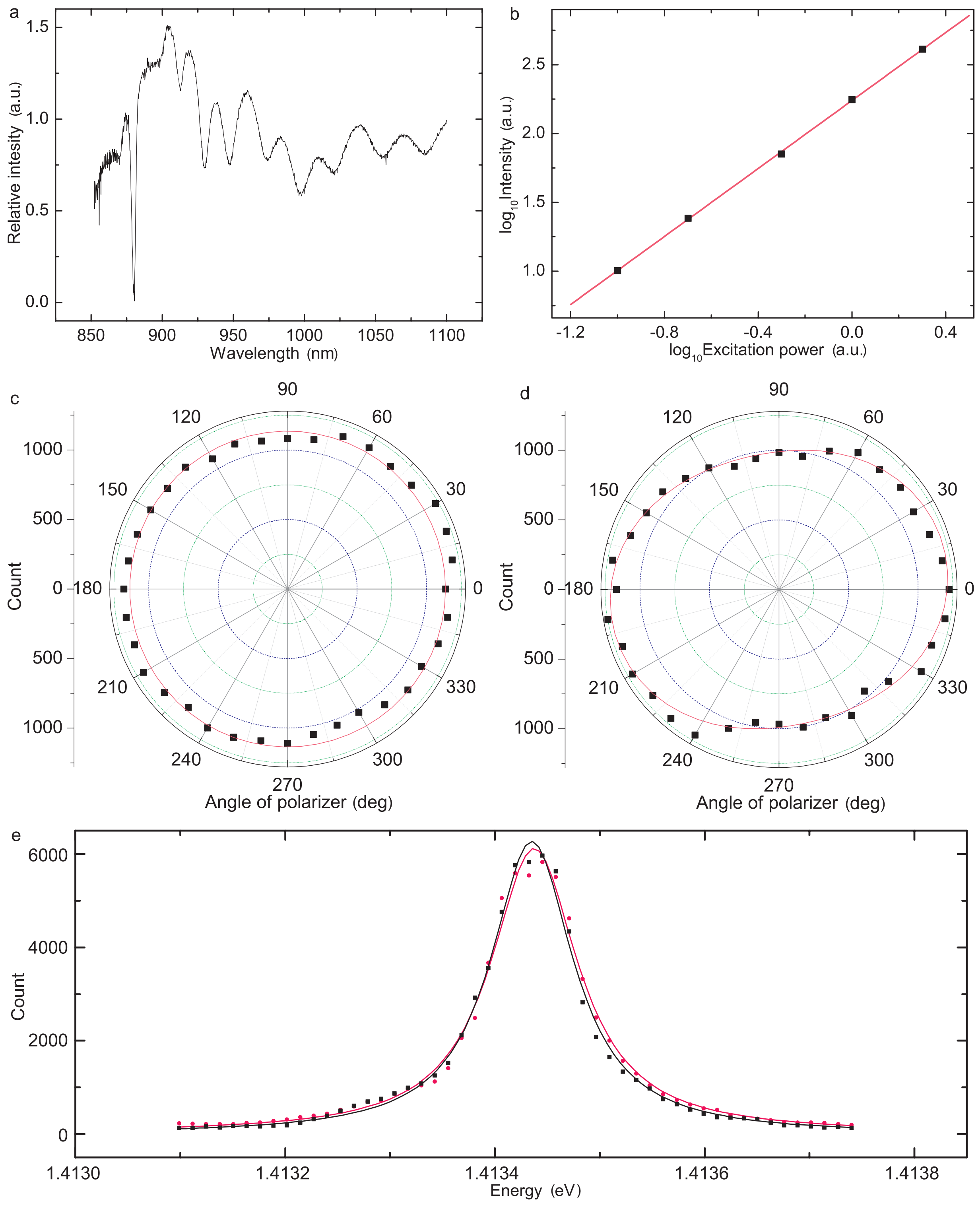

Supplementary Figure 2: **Studies of the QD's fluorescent characteristics.** (a) The reflective spectrum of DBR microcavity. The narrow dip at 880 nm is cavity mode of the DBR microcavity. The FWHM of this dip is 3 nm, which corresponds to the cavity Q value of 293. (b) Logarithmic plot of the intensity versus excitation power for the peak in Figure 2(a) in main text. The slope is fitted to be 1.235±0.011. (c, d) The polarization dependent measurement of the QD emissions without (c) and with (d) a QWP inserted before the polarizer. The pink lines are the theoretical fittings, respectively. (e) The polarization-resolved PL spectra of the QD. The black dots are obtained when the polarizer is set to the direction of π_x, and the black line is the Lorentz fit of this spectrum. Similarly, the pink dots are obtained when the polarizer is set to the direction of π_y, and the pink line is the corresponding fit. These two spectra coincide to each other well.

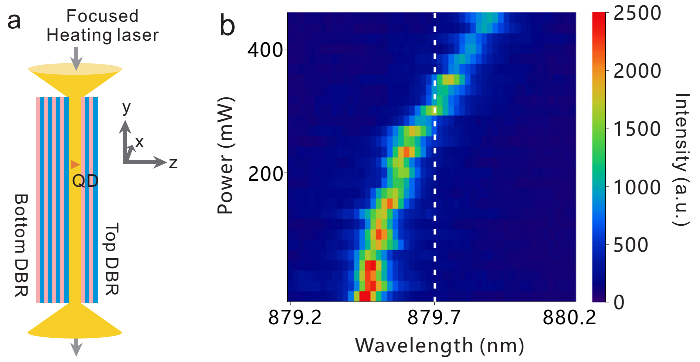

Supplementary Figure 3: **The local heating.** (a) The localization of QD area. This is the side view of the QD sample. The heating laser is focused on the cleaved edge of the sample via a long working distance objective. The DBR mirrors here play the role of a waveguide for the heating laser and direct this laser light to the QD to heat the vicinity of the QD. (b) Power-dependent spectra using the 1550-nm laser. The power of the 1550-nm heating laser should be much larger than that of the 910-nm laser. 300 mW is required to shift the peak to 879.7 nm.

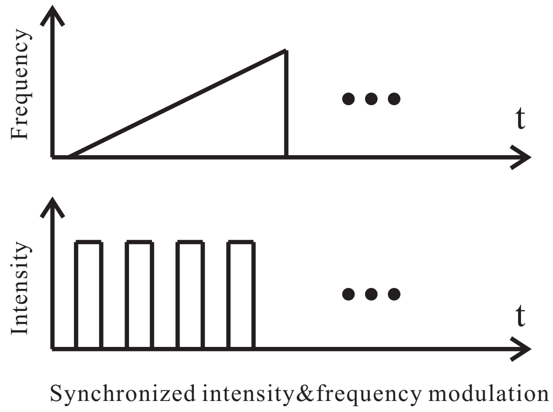

Supplementary Figure 4: **The modulation of pump laser.** The intensity of the pump laser is modulated by an AOM, which simultaneously shifts the laser frequency continuously.

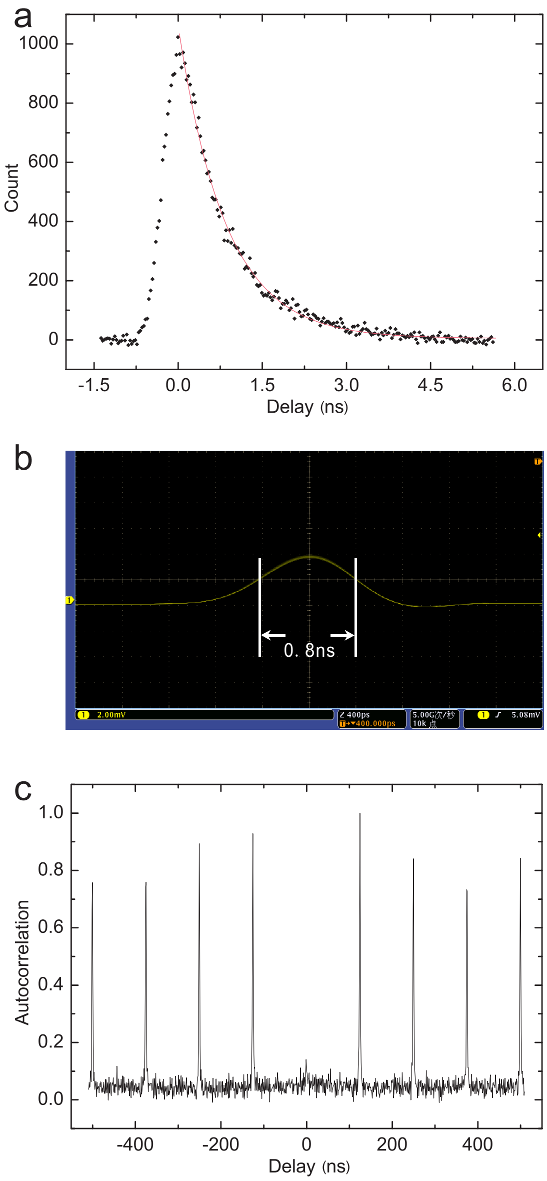

Supplementary Figure 5: **The single-photon characteristics.** (a) The lifetime measurement. The QD is excited using a 76-MHz picosecond pulse laser, and the data are collected and analyzed by a PTA. We use the exponential decay function to fit these data. The pink line is the fitting result, and the QD's lifetime is 0.849 ± 0.011 ns. (b) The temporal width of the excitation pulses. The yellow line is a measurement of the excitation laser chopped by an EOM driven by a series of fast electric pulses, using a fast photodetector and a fast oscilloscope. The pulsewidth is 0.8 ns. (c) The HBT experimental result. The missing of the zero-delay peak confirms there is only one photon in each pulse.

SUPPLEMENTARY NOTES

Supplementary Note 1

The quantum dot sample. The quantum dot (QD) sample is prepared on a semi-insulating (100) GaAs substrate with a VEECO Gen-II solid source molecular beam epitaxy (MBE) system. As illustrated in Supplementary Figure 1, the structure consists of a distributed Bragg reflector (DBR) with 32 periods of $\lambda/4$ $Al_{0.9}Ga_{0.1}As/GaAs$ as the bottom mirror, a 4λ-thick cavity in the center, and 10-periods $Al_{0.9}Ga_{0.1}As/GaAs$ DBR as the top mirror. An InAs QD layer with an ultra-low density of approximately 10^8 cm^{-2} is at the center of the 4λ planar cavity. This QD layer is grown 10 nm above the $Al_{0.2}Ga_{0.8}As$ layer to blue shift the emission wavelength to approximately 879.7 nm. Note that there are other ways to tune the emission of QDs to 879.7 nm, for example, using AlGaInAs QDs [1] or nonlinear frequency conversion (35% to 40% external conversion efficiency) [2]. We just have used the method that is more suitable for our experiment.

Supplementary Note 2

The photoluminescence spectrum. As shown in Figure 2(a) in the main text, there is a single peak in the photoluminescence spectrum. Two reasons may explain this result. One reason may be that the other peaks are filtered or weakened by the DBR microcavity. The reflection spectrum of the DBR microcavity is shown in Supplementary Figure 2(a). To derive this spectrum, a thermal light source with a wide spectrum is used to irradiate a mirror and the sample, respectively, and two spectra are obtained. Next, the latter spectrum is divided by the former spectrum to obtain the relative-intensity spectrum. In this figure, the background is subtracted. We find that a sharp dip appears at the position of 880 nm. This dip shows the cavity mode. When the light is resonant with the cavity mode, it is more likely to transmit through the DBR cavity, resulting in the dip in the reflected signal. The full width of half maximum (FWHM) is 3 nm, so other peaks are very likely to be weakened, whereas this peak will be definitely strengthened by the DBR cavity because of the improvement of the collection efficiency.

Supplementary Figure 2(b) shows the logarithmic plot of the intensity versus excitation power for this single peak. The pink line is the linear fit for the data. The slope is fitted to be 1.235 ± 0.011. According to the rate equations of the carriers in QD, the populations of the exciton and biexciton will increase linearly and quadratically, respectively, as the excitation power increases. Therefore, the slope should be 1 for the exciton and 2 for the biexciton. The intermediate value indicates that this peak is probable to be a trion [3–5]. This may be the second reason because the exciton line and biexciton line always appear as a pair, but the trion could have only a single line. The reason for this behavior is that there are two pairs of hole-electrons in a biexciton but only one pair in a trion (and an additional electron or hole). In the experiment, the generation of the trion is possible. Because the mass of the hole is much larger than that of electron, when they are excited, the electron moves faster than the hole. This unbalanced carrier capture enables a trion to be produced more easily.

We also perform the polarization dependent emission measurements for this QD using a quarter-wave plate (QWP) and a polarizer. The polarization state of the trion emission is supposed to be a mixture of $|\sigma^{\pm}\rangle = \frac{1}{\sqrt{2}}(|H\rangle \pm i|V\rangle)$, where $|H\rangle$, $|V\rangle$ denote the horizontal and vertical polarization states, respectively. Therefore, when only a polarizer is used in the measurement, the polarization spectrum is calculated to be a constant A; and when a QWP is inserted before the polarizer, the polarization spectrum is derived as $A_1 cos^2(\theta - \theta_0) + A_2 sin^2(\theta - \theta_0)$, with θ representing the polarizer's angle and θ_0 associated to the QWP's angle. The experimental results of these two situations are shown in Supplementary Figure 2(c) and Supplementary Figure 2(d), respectively, which are then well fitted with the corresponding theoretical formulas. The fitting results show the ratio A_2/A_1 to be 0.801 ± 0.015.

Furthermore, we measure the polarization-resolved photoluminescence (PL) spectra of this peak using a linear polarizer and a piezo driven etalon with a free spectral range (FSR) of 300 GHz and a bandwidth of 8 GHz. The results are shown in Supplementary Figure 2(e). The black dots and the pink dots correspond to single-photon emissions detected when the polarizer is set to the directions of π_x and π_y (the definitions of these notations can be referred in [6]), respectively. If the QD contains an exciton or a biexciton, the exchange interaction will cause a fine structure splitting (FSS) between these two polarization-resolved spectra; whereas, if the QD contains a trion, the exchange interaction will vanish, so the FSS will also vanish [6, 7]. Here by fitting our results respectively with Lorentz function [8] (the black and pink lines in the figure corresponding to the data represented with the same-color dots, respectively), we derive the difference of the centers of these two polarization-resolved spectra, which is 2.277 ± 1.383 μeV. This value is much less than the FSS reported in the previous works (typically tens of μeV to greater than 100

μeV) [6, 7, 9]. Therefore, to some extent we can conclude that our QD contains a trion within the accuracy of the experiment. The non-vanished value of the splitting may be caused by the imperfect optical mirrors and windows of the cryostat.

Although we suppose that our QD may contain a trion, we note that this is not an essential issue in this work. Exciton, biexciton and trion are equivalent here since they all emit true single photons. With this property at hand, reliable quantum repeaters can be constructed [10]. Furthermore, when specific carriers are used in our configuration, more advanced quantum applications will be realized.

Supplementary Note 3

The local heating. Supplementary Figure 3(a) shows a sketch of how the heating laser is directed to the vicinity of the selected QD (side view of the sample). First, the laser is focused to the cleaved edge of the QD sample using a 50X long working distance (20 mm) objective with N.A. of 0.42. Next, the objective is moved along the z axis until the DBR microcavity is found. In this situation, the DBR mirrors acts as a waveguide for the heating laser with the z direction restricted [11]. The separation between these two DBR is approximately 1 μm. Meanwhile, the dispersion in the x direction is estimated to be less than 0.4 mm due to the high refractive index (approximately 3.3) of the sample and considering the height (along y axis) of the sample of 3 mm. Next, we move the objective along x axis to find the selected QD with the wavelength-shift effect becoming closer to optimal. Finally, the distance between the objective and the sample in y direction can also be tuned slightly to make the focused spot be closer to the QD. Notably, other QDs exposed by this laser are also heated, but because the volume of the heating area is very small, the global sample remains cool; therefore, the inconvenience caused by heat expansion can still be avoided.

In this experiment, a high-power 910-nm laser was used as the heating laser, which has a photon energy below the bandgap of GaAs. However, the sample does not appear absolutely transparent for this light. There exists a weak absorption which may be due to the imperfections of the material of the sample, for example, the defect centers generated during the sample growth, which can absorb the heating photons and convert the energy into phonons. This phenomenon also appears in other systems, for example, the absorption of 1000 $\sim$ 1600-nm light in a silicon spherical microcavity [12]. In our situation, the laser power is high (24 mW); therefore, we can observe the heating effect clearly. We also use a 1550-nm laser to repeat the local-heating process, with the result shown in Supplementary Figure3(b). We find the power of the laser should be much larger than that in the 910-nm case. Approximately 300 mW is required to shift the peak to 879.7 nm, whereas the ratio of the coupling efficiencies to the DBR waveguide for the 910-nm and 1550-nm lasers is estimated to be not more than 2 : 1. This result can be explained by the fewer defect centers that are able to be excited by the 1550 nm photons because of the smaller photon energy.

There are other technologies to finely tune the wavelength of the QD's emission, such as electric field, strain, or magnetic field. Compared with these methods, local heating is more suitable for us. The first reason is that local heating is simple. Only a high-power laser is required, without the complex sample fabrication procedures, the requirement of piezoelectric actuators placed in the cryostat, or the use of a superconducting magnet. The second reason is the DBR mirrors that are used for improving the collection efficiency of single photons are just right used as a waveguide for the heating laser to flow to the selected QD.

Supplementary Note 4

The preparation of the atomic frequency comb. The atomic frequency comb (AFC) technique is utilized to realize reversible quantum state transfer between single photons and atomic excitations [13, 14]. The AFC protocol requires a tailored absorption profile composed of a series of periodic and narrow absorbing peaks separated by Δ. The input single photons can be absorbed and diffracted by the AFC. A collective echo emission is retrieved after a storage time $T_{\text{storage}} = 1/\Delta$ in the crystals.

The pump light for establishing AFC comes from a frequency stabilized Ti:sapphire laser (MBR-110, Coherent). Using the double-pass configured acousto-optic modulator (AOM2 in Figure 1 of main text), the pump laser frequency is swept over 100 MHz in each 200 μs cycle, and its amplitude is modulated periodically to give a comb structure (Supplementary Figure 4). This pump sequence is continuously repeated in the preparation phase to produce an optimized AFC structure. The AFC bandwidth prepared with the AOM is approximately 100 MHz and is limited by the effective bandwidth of the double-pass configured AOM. To further extend the memory bandwidth, the output light of the AOM is directed into a fiber-coupled high-speed electro-optic phase modulator (EOM in Figure 1 of the main text, where the fibers are not sketched) that creates first and second-order sidebands [15]. The comb at the

carrier frequency is copied twice on each side. To ensure zero frequency detuning for the copied combs, the driver frequency for the EOM is chosen as 100 MHz. The radio frequency (RF) power for driving the EOM is experimentally optimized to optimize the storage efficiency. In this way, an overall comb width of approximately 500 MHz is achieved in our experiment. During the 11.5-ms preparation time, the pump laser is sent to the Nd^{3+}:YVO_4 crystals to prepare the AFC by pumping ions with certain frequencies to the auxiliary state (see the inset of Figure 1 in the main text). The lifetime of the ground state and the auxiliary state is 42.9 ms, much longer than the 10-ms storage-and-retrieval time. This feature ensured that the single photons can be successfully stored in the storage-and-retrieval procedure when the pump laser is turned off in order to reduce the pump noise.

Supplementary Note 5

The spectral, temporal and photon correlation characteristics of the input and output photons. Here we primarily discuss the situation of narrow single-photon pulses, which attracts more interest in this work. The linewidth of the QD emission is approximately 25 GHz, which is determined using a piezo tunable etalon with a FSR of 300 GHz and a bandwidth of 8 GHz. The lifetime of this emission is 0.849 ± 0.011 ns, which means its natural linewidth is less than the detected linewidth. This lifetime is derived by using a picosecond pulse laser to excite the QD, and a picosecond time analyzer (PTA) to obtain the time spectrum, which is then fitted with the exponential decay function (see Supplementary Figure 5(a)). The line broadening was mainly caused by spectral diffusion. Resonant excitation can be used to reduce the linewidth in the future works (At best, 7-MHz linewidth of the QD emission has been achieved using resonant excitation [16], which is much narrower than the memory bandwidth here). After passing through the filters and the etalons (see Figure 1 in main text), the linewidth of the single photons is reduced to 700 MHz, which is determined by the bandwidth of the etalons. The memory bandwidth is 500 MHz; therefore, the linewidth of the retrieved single photons is limited to 500 MHz. The memory bandwidth is possible to be extended to several GHz by using one AOM and more EOMs to prepare the AFC. The temporal widths of the input and output photons can be derived by fitting the first peak (transmitted photons) and the second peak (stored photons) in Figure 4(a) in main text, respectively, with Gaussian function. The fitting results are 1.478 ± 0.128 ns and 2.400 ± 0.081 ns for the first and second peaks, respectively. The temporal width of the stored single-photon pulse is approximately 1.6 times of that of the input pulse. This ratio is approximately equal to that of the linewidths of the input photons over the output photons (700 MHz/500 MHz= 1.4).

Supplementary Figure 5(b) shows the screenshot of a fast oscilloscope (Tektronix, DPO4104B) with a resolution of 80 ps. The yellow line is the signal of the excitation laser detected by a fast photodetector (Thorlabs, DET02AFC) with 1.2 GHz bandwidth and 50 ps rise time. The excitation laser is modulated to narrow pulses using a 10 GHz EOM driven by a synchronized high-speed pulse generator (Tektronix, AWG7082C), which supplies a series of 0.5-ns electric pulses. The FWHM of the excitation pulse is measured to be 0.8 ns (the difference may be caused by the imperfect cables), which is less than the QD's lifetime of 0.849 ± 0.011 ns. Therefore, the QD can be excited only once per pulse, i.e., only one photon exists in the pulse. The Hanbury Brown-Twiss (HBT) experiment also illustrates this point, as shown in Supplementary Figure 5(c). During this HBT experiment, the electric pulses are supplied with the temporal width of 0.5 ns (as it is in the memory experiment) and the period of 125 ns. The integration time is 16.6 hours. The missing peak at the zero delay demonstrates there is only one photon per pulse. The autocorrelation is estimated to be 0.14. In the output pulses, there will also be one photon per pulse at most because the memory process will not increase the photon number.

SUPPLEMENTARY REFERENCES

[1] Schlereth, T. W., Schneider, C., Höfling, S. & Forchel, A. Tailoring of morphology and emission wavelength of AlGaInAs quantum dots. *Nanotechnology* **19**, 045601 (2008).

[2] Ates, S. *et al.* Two-photon interference using background-free quantum frequency conversion of single photons emitted by an InAs quantum dot. *Phys. Rev. Lett.* **109**, 147405 (2012).

[3] Besombes, L., Kheng, K., Marsal, L. & Mariette, H. Few-particle effects in single CdTe quantum dots. *Phys. Rev. B* **65**, 121314(R) (2002).

[4] Rodt, S., Schliwa, A., Pötschke, K., Guffarth, F. & Bimberg, D. Correlation of structural and few-particle properties of self-organized InAs/GaAs quantum dots. *Phys. Rev. B* **71**, 155325 (2005).

[5] Gomis-Bresco, J. *et al.* Random population model to explain the recombination dynamics in single InAs/GaAs quantum dots under selective optical pumping. *New J. Phys.* **13**, 023022 (2011).

[6] Bayer, M. *et al.* Electron and hole g factors and exchange interaction from studies of the exciton fine structure in $In_{0.60}Ga_{0.40}As$ quantum dots. *Phys. Rev. Lett.* **8**2, 1748-1751 (1999).

[7] Bayer, M. *et al.* Fine structure of neutral and charged excitons in self-assembled In(Ga)As/(Al)GaAs quantum dots. *Phys. Rev. B* **6**5, 195315 (2002).

[8] Tang, J.-S. *et al.* Measuring non-Markovianity of processes with controllable system-environment interaction. *Europhysics Letters* **9**7, 10002 (2012).

[9] Ramirez, H. Y. *et al.* Optical fine structures of highly quantized InGaAs/GaAs self-assembled quantum dots. *Phys. Rev. B* **8**1, 245324 (2010).

[10] Sangouard, N. *et al.* Long-distance entanglement distribution with single-photon sources. *Phys. Rev. A* **7**6, 050301(R) (2007).

[11] Flagg, E. B. *et al.* Direct evidence of interlevel exciton transitions mediated by single phonons in a semiconductor quantum dot using resonance fluorescence spectroscopy. *Phys. Rev. Lett.* **102**, 097402 (2009).

[12] Garín, M. *et al.* All-silicon spherical-Mie-resonator photodiode with spectral response in the infrared region. *Nature Communications* **5**, 3440 (2014).

[13] de Riedmatten, H., Afzelius, M., Staudt, M. U., Simon, C. & Gisin, N. A Solid-state light-matter interface at the single-photon level. *Nature* **4**56, 773-777 (2008).

[14] Afzelius, M., Simon, C., de Riedmatten, H. & Gisin, N. Multimode quantum memory based on atomic frequency combs. *Phys. Rev. A.* **7**9, 052329 (2009).

[15] Bussières, F. *et al.* Quantum teleportation from a telecom-wavelength photon to a solid-state quantum memory. *Nature Photonics* **8**, 775-778 (2014).

[16] Matthiesen, C., Vamivakas, A. N. & Atatüre, M. Subnatural linewidth single photons from a quantum dot. *Phys. Rev. Lett.* **1**08, 093602 (2012).
